# Using AI to Predict Patients' Length of Stay: PACU Staff's Needs and Expectations for Developing and Implementing an AI System

**DOI:** 10.1155/jonm/3189531

**Published:** 2024-11-14

**Authors:** Sara Lundsten, Maritha Jacobsson, Patrik Rydén, Lars Mattsson, Lenita Lindgren

**Affiliations:** ^1^Department of Nursing, University of Umeå, Umeå 901 87, Sweden; ^2^Department of Social Work, University of Uppsala, Uppsala 751 26, Sweden; ^3^Department of Mathematics and Mathematical Statistics, University of Umeå, Umeå 901 87, Sweden; ^4^Division of Industrial Doctoral School for Research and Innovation, University of Umeå, Umeå 901 87, Sweden

## Abstract

**Introduction:** The need for innovative technology in healthcare is apparent due to challenges posed by the lack of resources. This study investigates the adoption of AI-based systems, specifically within the postanesthesia care unit (PACU). The aim of the study was to explore staff needs and expectations concerning the development and implementation of a digital patient flow system based on ML predictions.

**Methods:** A qualitative approach was employed, gathering insights through interviews with 20 healthcare professionals, including nurse managers and staff involved in planning patient flows and patient care. The interview data were analyzed using reflexive thematic analysis, following steps of data familiarization, coding, and theme generation. The resulting themes were then assessed for their alignment with the modified technology acceptance model (TAM2).

**Results:** The respondents discussed the benefits and drawbacks of the proposed ML system versus current manual planning. They emphasized the need for controlling PACU throughput and expected the ML system to improve the length of stay predictions and provide a comprehensive patient flow overview for staff. Prioritizing the patient was deemed important, with the ML system potentially allowing for more patient interaction time. However, concerns were raised regarding potential breaches of patient confidentiality in the new ML system. The respondents suggested new communication strategies might emerge with effective digital information use, possibly freeing up time for more human interaction. While most respondents were optimistic about adapting to the new technology, they recognized not all colleagues might be as convinced.

**Conclusion:** This study showed that respondents were largely favorable toward implementing the proposed ML system, highlighting the critical role of nurse managers in patient workflow and safety, and noting that digitization could offer substantial assistance. Furthermore, the findings underscore the importance of strong leadership and effective communication as key factors for the successful implementation of such systems.

## 1. Introduction

The need for innovative technology in healthcare is apparent due to challenges posed by a lack of resources, including patient bed, staff shortages, and economic restraints [1–4]. These resource constraints can lead to stress among healthcare workers, limited patient throughput, and increase the risk of patient harm [5, 6]. It is therefore essential that limited healthcare resources are used efficiently. New technology can potentially aid in the efficient utilization of resources. Artificial intelligence (AI) is increasingly being recognized as a powerful tool in revolutionizing healthcare [7], particularly in areas such as efficiency and quality [8, 9]. This cutting-edge technology, especially in high-tech environments like the postanesthesia care unit (PACU), offers immense potential. AI's ability to generate predictions can significantly enhance patient workflow, optimize resources, and elevate the quality of care in the PACU, embodying the technological advancement and precision that these critical care settings demand [9]. However, although previous studies have shown significant potential, AI and its subarea, machine learning (ML), also pose challenges such as a lack of sufficient legislation [10], technical concerns, and ethical considerations [11–13]. Another challenge when building robust predictive ML models is the need for substantial amounts of high-quality data to train and build models. Fortunately, in hospitals, patients are closely monitored as a central part of PACU care. The monitoring process includes a variety of factors, including respiratory and cardiovascular functions, neuromuscular activity, mental status, body temperature, pain, and other relevant variables [14]. Each patient generates a vast amount of data, making it possible to build predictive ML models that can be used to predict patients' length of stay. This information can then be incorporated into a patient planning system.

In the PACU, nurse managers play a crucial role in coordinating patient workflow and overseeing the transition of patients from the operating room (OR) to the PACU and subsequently to a regular ward. This complex task requires not only the nurse manager but also all nurses in the PACU to possess exceptional skills, acquired either through specialist degrees or specific training. Their collective expertise is critical in quickly evaluating and predicting the level of care and treatment time necessary for each patient. This involves determining whether a patient requires a shorter postoperative stay with less monitoring or needs more comprehensive postoperative care and a prolonged duration in the PACU. Such specialized skills ensure that every patient receives individualized care, tailored to their unique recovery requirements [15]. The focus on individualized patient care involves not only monitoring vital signs but also early identification of the potential complications that may arise after surgery and anesthesia [16]. This comprehensive approach is vital for effective management of patient recovery in these advanced care settings. However, it also accounts for the dynamic nature of both patient workflows and nurse workloads, which can fluctuate significantly from 1 h to the next. The variability in the number of patients and their care needs and unexpected changes in staffing pose a significant challenge in planning postoperative care and optimizing resources [15]. Moreover, a PACU operating at full capacity with minimal staff not only intensifies this challenge but also increases the risk of postoperative complications and the likelihood surgeries will be canceled [17].

Such scenarios are not only devastating for the patients involved but also carry substantial health and economic repercussions [5], highlighting the critical need for efficient and adaptable postoperative management strategies. Systems built upon ML predictions appear to have great potential when it comes to addressing these issues [18]. The transition from research and pilot testing of ML systems in healthcare to actual implementation remains slow [19–22]. Recent studies in the PACU show promise in predicting unplanned care escalation, where patients unexpectedly require a higher level of care [23]. In addition, Tully and colleagues [24] have demonstrated encouraging results in predicting the risk of prolonged stays in the PACU. Levin et al. [25] found that ML models for discharge planning can improve patient flow in hospitals. Despite these promising findings, the successful implementation of ML systems in complex environments like the PACU has been limited. However, the technology is advancing rapidly, with an increasing number of successful healthcare implementations being reported [26, 27]. Current literature does not extensively document or explore the implementation of advanced technology capable of making *near real-time, individual predictions* in a way that is beneficial for healthcare staff. Additional research is needed to understand the successful implementation of these systems. In addition, previous studies emphasized the need for nurses to engage in the development and implementation of ML in healthcare [28]. Furthermore, it is important to understand users' acceptance and adoption of new technology, and to acquire this understanding, the technology acceptance model (TAM), a theoretical framework developed by Davis in the 1980s [29], may prove beneficial. TAM has previously been shown to be a valuable tool for understanding how users come to accept and use new technology. It suggests that users' intention to use a technology is influenced by two main factors. The first of these is perceived ease of use (PEOU), referring to the user's perception of how easy it is to use the technology. If a user believes that a system is user-friendly and easy to learn, they are more likely to accept and use it. The second factor, perceived usefulness (PU), refers to the user's perception of how useful the technology will be in their work or daily life. If users believe that a system will enhance their productivity or help them achieve their goals, they are more likely to accept and use it. An extended version of TAM was introduced in 2000 [30]—TAM2—which provides a more comprehensive understanding of technology acceptance by taking into consideration not only the individual's perception of ease of use and usefulness but also the influence of external factors and motivations. TAM2 also includes the concepts “social influence processes” (subjective norm, voluntariness, and image) and ““cognitive instrumental processes” (job relevance, output quality, result demonstrability, and PEOU). TAM2 has been applied in healthcare to address factors influencing technology adoption [31, 32]. Understanding these factors is important for evaluating technical solutions in healthcare. To explore issues surrounding technology acceptance and adoption, we wanted to investigate PACU staff's needs and expectations as part of our process developing and implementing an ML-driven system for predicting patient length of stay in the PACU. A qualitative approach was taken and a reflexive thematic analysis (RTA) was adopted; this has previously been described in research areas considering healthcare [31, 33]. Semistructured interviews were conducted with staff managing patient workflow, both pre- and postoperatively.

## 2. Aim

The aim of the study was to explore staff's needs and expectations concerning the development and implementation of a digital patient flow system based on ML predictions.

## 3. Methods

This qualitative interview study included staff members with various roles involved in planning perioperative patient workflows at the largest university hospital in northern Sweden. The hospital has a catchment area that covers nearly half of Sweden and treats approximately 25,000 in-patients yearly. In the introductory section, respondents were presented with a hypothetical scenario that outlined the potential applications, conceivable usage possibilities, and technical prerequisites for the proposed ML system. The interviews were held individually in a semistructured manner and were recorded, transcribed, and analyzed according to RTA [34]. The interviews were securely stored in a decoded format within a restricted file area at Umeå University, accessible only to the researchers.

### 3.1. Proposed ML System

The proposed ML system will be built using patient data from a database containing patient records. Respondents received a verbal introduction to the proposed system during the interview. Since none of the participants had prior knowledge of ML models, a hypothetical scenario was presented to explain how these models are developed, how they make predictions, and how the results would ultimately be presented to the end user.

### 3.2. Study Design

The study utilized semistructured interviews to gather staff perspectives on diverse issues related to the planning and introduction of new technologies, such as intervention, planning, organizational and structural settings, and staff concerns, demands, and reflections. Interview questions were constructed to capture the complexity of ML system implementation in healthcare settings. The objective of the interviews was to explore multiple perspectives and factors that affect the development, adoption, and implementation of new technologies within an organization.

#### 3.2.1. Setting and Timing

This study was timely, overlapping efforts by the Swedish government and by local politicians advocating for digital transformation in healthcare. Therefore, our study occurred within a supportive context reflecting the broader trend toward embracing digital solutions in healthcare. The study was conducted in the region's largest hospital, which had been an early adopter of electronic medical records (EMR) use in perioperative settings. This early adaptation facilitated the utilization of the extensive data available in the clinic's database, which houses a considerable volume of data from the PACU, where approximately 10,000 patients are treated annually.

In the hospital in question, the PACU has the capacity to provide care for 22 nonintubated patients of all ages simultaneously and is staffed around the clock throughout the year, ensuring continuous availability of medical personnel. When this study started, patient workflow planning at the PACU was performed manually. Using pen and paper, a flowchart was drawn, estimating when patients are arriving and leaving the PACU. The flowchart is manually synchronized with the staff's work schedule and staff are stationed at specific bed locations. All changes throughout the day (staffing or patients) are manually updated. The flowchart is needed to ensure that there is room for all planned patients, since patient length of stay can vary from 1 h to several days. Manual planning requires professional knowledge and experience in surgical procedures, and at times relies on guesswork.

#### 3.2.2. Respondents

The goal of the recruitment process was to acquire a representative sample of staff involved in patient flow management perioperatively, as it has a direct impact on patient throughput. To achieve a representative sample of staff, management was requested to select staff members from a diverse range of professions and levels of experience. None of the invited respondents declined participation. Their experience in their current occupation ranged from 2 months to 31 years, with a mean of 11 years. The 20 respondents included 10 nurses or physicians in managing or leadership positions and 10 nurses primarily working closely with patients. The occupations of the respondents invited to participate in the study included nurses working in the PACU, nurses working in the OR, managers responsible for staff planning, managers responsible for planning patient workflow, attending physicians, surgical schedulers, and physicians and nurses with administrative roles. The study followed ethical guidelines and national laws [35]. All 20 respondents were given oral and written information about the study and their right to withdraw from the study at any time during the research process. The study was approved by the Swedish Ethical Review Authority Ref. no: 2021-06140-01. All respondents completed their participation, and the recruitment process was successful in achieving its goal of obtaining a representative sample of staff members involved in patient throughput management perioperatively.

#### 3.2.3. Data Collection

Interviews were conducted from April to December 2022. All interviews took place near the respondents' workplaces and were conducted by the same member of the research team using a custom-designed interview guide (Appendix 1) to ensure consistency throughout the process. The interviews were conducted in Swedish by the interviewer who was a trained specialist nurse with extensive experience in both the technical and clinical aspects of the field.

The interview comprised narrative and open-ended questions (e.g., tell me what you consider to be key factors for success when implementing new technology), and follow-up questions were applied when needed. Interviews were audio-recorded and transcribed. The interviews varied from 19 to 65 min (with a mean of 41 min).

#### 3.2.4. Data Analysis

We applied a qualitative approach when analyzing the interviews. The analysis was inductive and grounded in the existing material. However, considering the research team's prior knowledge of the workplace setting and its information system, along with theoretical perspectives, the analysis incorporated both inductive and deductive elements. The analysis was inspired by RTA proposed by Braun and colleagues [34]; which means that analyses are based on the researchers' subjective and reflexive interpretations as well as theoretical flexibility. An advantage of this type of analysis is that it is flexible and does not require detailed theoretical knowledge [34]. Themes are actively created by researchers both inductively (i.e., bottom-up) and deductively (i.e., top-down).

The material was coded in relation to underlying and semantic meanings. Together, the researchers reflexively discussed and analyzed the material (Table 1). Both the transcripts and the recordings were read and individually listened to several times. Relevant citations were marked in the texts and then clustered in color-coded themes. Theoretical preconceptions and earlier research were discussed, and researchers took notes throughout the entire research process. Gradually, themes emerged from the texts from the researchers' shared interpretations [34]. According to Nowell et al. [36], thematic analysis is a flexible method used to explore the respondent's perspective and to fill knowledge gaps in “how to” successfully develop and implement advanced digital systems in healthcare settings. Finally, to gain a deeper understanding of the user's acceptance and adoption of new technology, we assessed the themes (Table 2) concerning their alignment with the TAM2.

It is important to note that RTA is not a linear process; researchers frequently move back and forth between phases as their understanding of the data deepens and as themes are developed, reviewed, and refined. RTA emphasizes the active role of the researcher in the analysis process, acknowledging that the identification of themes is shaped by the researcher's judgments, insights, and interactions with the data [34].

#### 3.2.5. Rigor

This study used several strategies to reinforce the validity of its results, including adherence to the standards for reporting qualitative research (SRQR) and the consolidated criteria for reporting qualitative research (COREQ) guidelines, which were followed throughout the entirety of the study. Furthermore, the interview guide was constructed to capture a broad understanding of the respondents' complex work environment. All interviews were transcribed by a professional transcriber, and the research group verified the accuracy by repeatedly listening to the recordings. Respondents were selected to reflect the ward's staff regarding representation in terms of profession, experience, age, and gender.

## 4. Result

Respondents discussed both the benefits and drawbacks concerning AI's potential to improve the planning system. They were asked to describe and reflect upon their current manual planning of patient flow through the OR and PACU. In the PACU, this was performed with pen and paper mainly by a highly experienced nurse manager. Patient flow is unevenly distributed throughout the day, characterized by both peaks and valleys, rendering the PACU work environment occasionally complex and demanding. When respondents considered the benefits and drawbacks both in the manual planning and the proposed ML system, most of them expressed a positive attitude regarding the transition to the proposed ML system. Four themes were identified: (1) controlling PACU throughput, (2) prioritizing the patient, (3) communication strategies, and (4) adapting to new technology. The benefits and drawbacks related to each theme are presented in Table 2.

### 4.1. Controlling PACU Throughput

Respondents reflected on the workflow and described manual planning as something that undergoes frequent changes in patient flow throughout the day. Most of them expressed frustration and pointed out inefficiencies with manual planning but also recognized it as being better than no planning at all.

It's detailed from the morning, but there are so many changes throughout the day, and everyone knows that, but it's still good to see what's planned initially. The downside of the planning being on paper is that we work in multiple different rooms, and keeping up with the communication between them is a challenge. Then comes erasing and writing on the paper, using correction fluid. It gets harder as the day progresses, especially by the afternoon, to keep that overview, which patients have arrived and who has left the neighboring room? And that's for both or all three rooms /…/ But it's busy everywhere, so you don't always have that overview. Then you must go into the patient record and check as well. You must access a computer system to see, but you don't know who just arrived and who is about to leave from the other side of the wall (PACU nurse 5).

Because it's often an irritation that you need to find space and staff, every day with every patient you come there with, so you never know /…/ As it is currently, it's so inefficient it's unbelievable (Nurse Anesthetist 18).

The manual planning system was described by respondents as complex and difficult to master. They expressed that it made the work situation challenging due to poor visibility of the workflow, as the information was restrained to physical paper documents. Moreover, manual planning was also expressed as vulnerable because it relies on healthcare personnel's individual experience to estimate a patient's care duration, with only a limited number of individuals consistently involved in patient planning.

Patient planning is also quite tied to our nurse manager, I would say. When she's away, things don't run as smoothly, especially if we have a lot of patients /…/ It's difficult (planning), and since it's difficult, I think it has become a bit person-dependent (PACU Nurse 8).

The proposed ML system was believed to be beneficial for supporting patient flow and occupancy management in real time. An important aspect that emerged during the interviews was that a future ML system needed to support the ability to control patient flow and occupancy. To achieve this control, they expressed a desire that changes made in the new system should be clearly visible in a lucid way and easy to access for all involved staff. Furthermore, they expressed concerns about what would happen if complete control was transferred to an ML system. They believed that there would be substantial opportunities for improvement if they retained the ability to operate manually within the system. Another concern mentioned was the risk of technology making miscalculations, but on the other hand, they also reflected on the fact that humans can make mistakes too.

No, I mean I just think that development is positive with new technology. So, I only see that it could get better [laughs]. But it's the machine that's supposed to figure it out and it might be wrong (miscalculation of predictions) sometimes /…/ I wouldn't think too much about it, because such things happen now too, with these paper slips we have, when it is people sitting and deciding (PACU Nurse 4).

Concurrently, respondents acknowledged the advantage of fostering a shared understanding among all staff to enhance control. They perceived that the proposed ML system should be a robust system and offer a long-term perspective on scheduling surgeries and the ensuing postoperative care. They noted the complexity of long-term planning, given the involvement of multiple individuals or clinics, each with visibility only pertaining to their respective domains and not the broader picture. As one manager (11) expressed, “Everyone sees the same information all the time, and that gives a common picture.”

Respondents saw the potential for an AI system to enhance trustworthiness and safety through its ability to predict care duration and handle large volumes of information.

The more it is considered, the safer it becomes, so I can't say that this is not suitable, because all information that we can assess is good information (Manager 13).

In summary, the respondents talked about the benefits of introducing the proposed ML system when it comes to controlling PACU throughput, above all at the organizational level. They expected that it would increase the reliability of predicted patient lengths of stay, demonstrate robustness, provide user-friendly access, and ensure clarity for users. At the same time, there was some concern about what could happen if people were not involved in the ML system, since it lacks qualities such as intuition and empathy. This perspective can be linked to the TAM2 framework [30]: the expected improvements in reliability and user-friendliness align with TAM2's constructs of PU and PEOU. Meanwhile, the concerns about the system's lack of human qualities like intuition and empathy touch upon the subjective norm aspect of TAM2, highlighting the importance of human oversight and involvement in technology adoption.

### 4.2. Prioritizing the Patient

The importance of prioritizing patient's needs was strongly voiced by all respondents. They highlighted a key risk for potential patient harm due to overcrowding, which happens in the manual planning system. Overcrowding in the PACU arises when the influx of patients exceeds the capacity of available staff and equipment. This situation often occurs from a lack of real-time visibility into patient flow across the surgical pathway, hindering effective coordination and resource management. In case of overcrowding, there is a risk that patients could not be monitored postsurgery.

And then I must be a bit ahead and try to plan so that we can accommodate all the patients when they're on their way from surgery. And it's not always easy, because surgeries that are supposed to take longer can suddenly be shorter. And it can even happen that there's no space, no floor area, and then you can ask for help from the doctor anyway and see how… “Can we discharge any patient earlier?” Or how we can make it work because the (patient) flows are affected by not wanting to stay too long either. You should stay for the time you need care after surgery, and then you need to move on to the ward, when it's stable and you have met the discharge criteria; otherwise it gets too crowded (PACU Nurse 5).

Respondents working at PACU expressed that they wanted to spend more time with patients. However, referring to manual planning, they described that it was difficult and time-consuming to search for information in several different systems while simultaneously caring for patients.

It's difficult to keep track of all the systems at the same time and take care of patients (PACU Nurse 5).

There was a hope from the respondents that the proposed ML system would allocate additional time that could be utilized in patient-focused care at the PACU. This time was seen as an opportunity to use for the preparation and optimization of the patients, as they no longer needed to focus on planning patient flow.

We will be able to focus more on the patients with this, and that feels really great. We won't need to keep track of that part (PACU Nurse 2).

Positive expectations were expressed by respondents regarding the proposed ML system, but they also discussed concerns related to patient safety. There was a fear that patient confidentiality might be compromised if this aspect was not considered during the development of the digital system. Moreover, a less experienced nurse voiced a concern that the predicted care duration dictated when patients would leave the postoperative unit. This belief contrasted with the opinions of more experienced individuals, who believed that patients' well-being should determine their discharge. Furthermore, more experienced nurses imagined that an ML system could offer reassurance to newly hired staff by providing guidance on the patients' care duration.

You see like a countdown on the computer like this, that this one is supposed to leave in 20 min, and you feel like that's not going to happen [laughs]. And maybe you get stressed by it and it feels like you're doing a bad job or something. It could turn out that way, if you don't see it as a tool, but rather as some kind of performance measurement, sort of. That this is the time you have, and you don't have any more time (Manager 9).

In summary, respondents expressed that the proposed ML system would save time on administrative tasks, enabling them to engage more directly with patients. These aspects show that the ML system has a direct relevance for job assignments and work quality, which are crucial aspects in getting humans to accept technology according to TAM2. In addition, experienced nurses also believed that the ML system prediction could serve as a guideline on patients' care duration, which could play a critical role in preventing bed overcrowding. This ability could lead to a direct improvement in efficiency and aligns with the TAM2 concepts of result demonstration and job relevance, important in encouraging users to adopt new technology.

### 4.3. Communication Strategies

Respondents expressed that there are problems obtaining and transmitting information with manual planning. One example they gave is that overreporting must take place orally, and in some cases written information is also required, which is time-consuming. Another problem highlighted was that the current operational procedures necessitated logging into multiple systems to access essential patient information.

Well, I can gather data about a patient, information like what their background is. And then I can also look at x-ray and lab test results and blood gas analyses and stuff like that. For instance, I can compare what my colleagues have done, I can check in the patient record and go back and see that, here the patient was in a lot of pain and then they (my colleagues) gave this (dose and drug), and it seemed to have had a good effect (PACU Nurse 4).

External actors, who needed access to planning and patient information, also experienced a deficiency in communication with the manual planning. They argued that certain information regarding the ongoing care of patients was not available digitally, but rather (human) communication/information exchange took place on-site.

So usually there has been no contact with the postoperative anesthesia care unit at all. You don't know which place to go to, or anything, really (Nurse Anesthetist 18).

Respondents also reflected upon the potential of the new digital system to facilitate increased time for communication and dialogue between staff. It was anticipated that the new system would provide adequate information, a prospect deemed by respondents to potentially free up time for other essential human interactions.

But if it's a reliable system, we also must learn to let go (of unnecessary tasks), if we don't need to do them. Then there are a thousand other things (to do). Maybe then we'll have even more time for (human) dialogue (Manager 11).

Regarding the benefits and drawbacks of interpersonal communication, there were different perceptions among respondents. Some expressed, as in the quote above, a hope that in the future they would have more time for interpersonal communication, while others posited that they would no longer need to communicate face to face, as all information concerning the patient will be available in the new digital planning system.

One of the advantages emphasized in terms of communication was that digital systems can interact with each other. Consequently, respondents stressed the importance of consolidating data from all these sources within the ML system to prevent the loss of critical information. This approach was seen as not only enhancing safety and quality but also as optimizing efficiency, ultimately saving valuable time.

They (the digital systems) will talk to each other, so in that way, we won't need to check in our surgical planning system (Manager 3).

I hope that I won't have to work in five different systems…with different logins (Manager 17).

Respondents also spoke about the importance of visual communication in the new system. They expressed that colors and symbols contribute to making information easier to obtain. Members of the operational staff saw clear benefits in the digital capability to “highlight” vital patient information, thus minimizing the risk of its oversight or loss.

Also, I imagine you could consider color codes, that there should be a color code directly for (e.g., blue color equals a child) “It's a child” (Manager 3).

May be patients haven't had a catheter and be aware, that they haven't urinated, for example, that it's been noted. There are times when that's one of those details that you want to be carried forward (Nurse Anesthetist 18).

Several of the respondents also expressed a desire for a user-friendly ML system with a focus on minimizing information overload. Two of them expressed it in the following way:

Yes, as mentioned, quickly comprehensible, and without too much duplicated information (Nurse Anesthetist 18).

Oh no, not another system, I can't take it anymore, it's like getting a digital overload (Manager 17).

In summary, there was a consensus that an ML system would likely enhance quality of care and efficiency by providing quick and clear access to relevant information for all stakeholders involved. These findings also align with TAM2, since this is highly job-relevant and demonstrates enhanced results and quality.

### 4.4. Adapting to New Technology

Most respondents were positive about the prospect of an ML system, but they acknowledged that some colleagues might not be equally convinced. Overall, as healthcare becomes increasingly advanced and complex, and respondents recognized the growing need for digital systems. One nurse manager even suggested that the adoption of new technology could boost the self-esteem and pride of the workgroup.

Because it might be very easy when working with digital systems, but most of us, and my older colleagues especially, aren't particularly computer-savvy. And then it can be a challenge because not everyone is interested in the new device and wants to learn it (PACU Nurse 5).

Some managers reflected on the importance of involving all staff when introducing new systems, emphasizing that it was crucial for the ML system not to be perceived as an additional task or burden.

So, transparency and, therefore, a willingness to educate when implementing it. Not withholding certain persons; I mean, everyone should be included, and everyone should feel involved (Manager 13).

To ensure successful implementation, most respondents stressed the importance of making the system user-friendly. They also emphasized the significance of receiving well-tailored training, allocating dedicated time during the system's introduction, and receiving support and guidance from management.

Understanding from management. Understanding and knowledge of what this will mean technically, timewise, that it might take longer. And that it's anchored all the way up, from top to bottom in the management system or hierarchy that exists. I think the fact that everyone is aware of what we are doing or what is expected of us is very important (Manager 13).

Personnel possessed prior experience navigating various digital systems, fostering awareness among respondents that the initial stages of implementing the ML system could present challenges. However, they believed that it would eventually facilitate their work. In a later stage, after implementation, the primary concern of staff members was that the technology might entail technological malfunctions, but several respondents already had strategies in place to address these issues as they arose.

After all, it's always, when there's something new that you must work your way in. Once you've worked your way in, it speaks to you. There is a lot that is clear. Then it can be some computer stuff… Sometimes it can be that you have been used to doing things on paper. Then you almost think it's faster [laughs], because it feels easier then and there (Manager 3).

In summary, respondents expressed confidence that the new technology would prove beneficial and user-friendly once implemented. Some of them acknowledged that their colleagues might encounter challenges adopting the new technology. Nevertheless, they also believed that with management's support and appropriate training, the system would quickly become robust. This sentiment aligns with the TAM2 framework, particularly in the domains of PEOU and PU. The anticipation of managerial support and training correlates with TAM2's emphasis on facilitating conditions, suggesting a comprehensive understanding of the factors that influence successful technology adoption and utilization.

## 5. Discussion

In exploring the respondents' needs and expectations for a future ML system in the PACU, four key themes emerged: Controlling PACU Throughput, Prioritizing the Patient, Communication Strategies, and Adapting to New Technology. These themes encapsulate the various dimensions of how an ML system could potentially reshape current practices and address existing challenges.

The respondents consistently highlighted the potential of an ML system to enhance Controlling PACU throughput. They emphasized that such a system could significantly contribute to increased trustworthiness, safety, robust planning, accessibility, and lucidity in the PACU workflow. Nurse managers described their roles in the current manual planning as chaotic, stressful, and difficult to manage due to frequent last-minute changes. This chaotic environment underscores the urgent need for an intelligent technical solution. Poor planning often results in a full PACU, leading to a stressful working environment, which has been shown to increase the risk of errors and potential patient harm, as noted by de Oliveira, Garcia, and Nogueira [37]. A robust planning is particularly important in a healthcare environment where the stakes are high, and efficient management is crucial. The positive attitudes expressed by the respondents are consistent with findings from previous studies [38, 39]. Maassen and colleagues reported that 70% of their participants had either a positive or very positive attitude toward AI in medicine, and Holzner and colleagues suggested that ML systems could bring substantial improvements in safety, quality and efficiency.

Prioritizing the patient was another critical theme identified in the discussions. Respondents reflected on the possibility of reducing administrative burdens through an automatic planning system, thereby allowing healthcare professionals to focus more on patient care. Given the current shortage of healthcare resources, this shift was seen as not only beneficial but also crucial, a perspective supported by findings in other studies [24]. The demand for innovative technical solutions to optimize healthcare delivery is growing, as reflected in the literature [2, 40], and the respondents' insights align with this trend. There is often concern that AI systems might replace human jobs [41, 42], potentially leading to resistance against such technologies. However, in the context of nursing care, specifically with the implementation of an ML-driven patient flow system, the opposite effect may be observed. By automating analytical and logistical tasks, the ML system allows nurses to devote more time to intuitive and empathetic patient care – an aspect of nursing that cannot be replicated by AI. This perspective also aligns with discussions in service-oriented roles, where AI is seen as augmenting rather than replacing human work [43]. Therefore, the proposed ML system was not viewed as a threat to nursing roles but rather as a tool that enhances the quality of care, similar to the result of previous studies [41, 42].

In terms of Communication Strategies, the respondents anticipated a shift toward more efficient digital communication solutions. Communication errors are a significant contributor to adverse events in healthcare. Poor communication can lead to misunderstandings, incomplete information transfer, and ultimately, medical errors that compromise patient safety [44]. The integration of ML systems into healthcare has the potential to significantly enhance communication and information management. By automating and optimizing the planning processes, ML systems can streamline the flow of information, reduce human errors, and facilitate more effective communication among healthcare professionals [40, 45].

The positive attitude and the potential benefits of the new ML system, as recognized by respondents, align with the TAM2 constructs of subjective norms and image [30]. Staff acceptance is influenced by peer perceptions of the technology (subjective norm) and the potential boost to their professional status (image). In addition, the staff's focus on patient care, despite expected changes in communication, underscores the job relevance aspect of TAM2 [30]. This suggests that staff perceives the ML system as relevant and beneficial to their work, increasing the likelihood of its acceptance and use. In addition, concerns about maintaining confidence in effectively controlling PACU throughput reflect on TAM2's “cognitive instrumental processes.” This suggests that staff are deeply aware of the quality of outcomes the ML system will deliver and the significant benefits it offers. These benefits could help address the challenges associated with implementing advanced technology in healthcare [18, 21, 46].

However, participants also highlighted concerns about the system if the proposed ML system was not under human control and stressed the risk of patient identities being disclosed. They also mentioned that even though they themselves, didn't have issues with new technical solutions, others might encounter technical difficulties when adapting a new system. Although respondents in this study mainly expressed positivity toward implementation, earlier research has shown that the implementation of AI-based systems can be challenging. A possible explanation for these challenges is a limited understanding of the needs of healthcare personnel [13, 21]. Svedberg and colleagues [13] further argue that the implementation of technology in healthcare often neglects the involvement of those who could benefit from it, potentially failing to understand its possible effects [13].

Under the theme Adapting New Technology, the respondents in our study discussed the importance of having management support, building team spirit, tailored education, and having user-friendly solutions when implementing the ML system. These results are comparable to those reported by Gyldenkærne and colleagues [47]; who emphasized that collaboration was a crucial element for the successful development and implementation of ML systems. Other key factors for a successful implementation included providing adequate training and resources. Gyldenkærne et al. also noted that a common obstacle to success was the lack of managerial support [47]. A recent report from the Swedish government emphasizes that a digital tool is simply a means to achieve the objective of providing safe and effective healthcare for citizens, encouraging staff to thrive without unnecessary risks [48]. Our results have encountered all these aspects to varying degrees.

Building on these insights and the findings from the current study, the research team has developed an ML system that has not yet been implemented but is planned for the next phase of the project.

### 5.1. Implications for Nursing Management

Nurse managers play a pivotal role in optimizing patient workflow, avoiding bottle-neck situations, and minimizing patient harm. The digitization of the healthcare sector offers several opportunities to aid nurse managers in their daily work [22, 49]. Respondents in our study identified several factors for successful implementation, such as the importance of strong positive leadership with clear communication. While nurse managers may not always hold the highest managerial position, they frequently occupy key roles in leading and planning daily work. Many nurse managers are responsible for control over daily work, and even when an AI system assists in decision-making, it remains vital to retain the ability to assume control and make new decisions if necessary. These findings show the importance of incorporating manual control features into the ML system. Communication was identified as crucial for a smooth workflow, and hence advanced systems need to help staff communicate with each other. Similar findings were found in 2015 by Kimbrough and colleagues, where they emphasized that optimizing efficiency in an OR context could be achieved through the introduction of technology that is both easily accessible and adaptable. Such technology should allow for configuration adjustments and enable rapid communication with relevant personnel. In 2015, this translated to the utilization of tools like magnetic whiteboards and text messages via cell phones [50].

The ability to develop a designated intelligent ML system based on nurse managers' and surrounding staff's requirements and wishes might have the potential to optimize resources, reduce stress, and enhance quality of care.

### 5.2. Limitations and Areas for Future Research

The current study set out with the aim of identifying key factors of importance for developing an ML system aimed at assisting in the daily planning of patient workflow in the PACU. While many key factors were identified, they might be limited to the PACU setting, and their applicability in other healthcare settings remains uncertain. Further research is needed to understand which factors can be generalized to a broad healthcare setting, given that patient flow management is relevant in many healthcare settings. Implementing advanced technology in healthcare is also a significant barrier to overcome [13, 51], and further research is needed to ensure successful development and implementation.

Having a single interviewer could be seen as a limitation, as different perspectives and details might be overlooked [52]. However, the interviewer possessed a deep understanding of both the technical and clinical aspects, which helped facilitate discussions on technical issues and provided valuable insights into the clinical context. The professional relationship between the interviewer and some participants may have led to more cautious responses to avoid affecting future collegial relationships. On the other hand, this familiarity could have fostered greater trust, resulting in more honest and detailed answers [53]. In addition, the interviewer was involved in the development process of the ML system, which might have influenced respondents' attitudes. However, it is worth noting that all interviews were conducted before development began, potentially strengthening the credibility of the current study. The respondents in the current study were mainly women, which might not reflect the needs and wishes of a male perspective. However, most healthcare staff members are women, making it appropriate to address their requirements and needs.

## 6. Conclusion

In healthcare, many tasks are manually executed, requiring highly skilled staff and strong, supportive leadership for effective workflows. Introducing advanced technology, like an ML-enhanced planning system, promises significant benefits. Our research indicates that for the successful development and implementation of such a system, it is essential to address several key factors that enable staff to effectively control PACU throughput and also prioritizing the patient, such as the need for a robust planning, an accessible system, and guidance on the patients' care duration. Furthermore, our findings suggest that while traditional communication strategies can be time-consuming, they can be optimized through digital communication. Successful adaptation to new digital systems and workflows requires management support, the cultivation of team spirit, tailored education, and the implementation of a user-friendly system. These factors are crucial for empowering staff to manage their responsibilities effectively. Successful development and implementation of such an ML system necessitates collaboration with healthcare staff, focusing on their needs, desires, and expectations.

## Figures and Tables

**Table 1:** Overview of the analysis process.

Familiarization with the data	The interviews were professionally transcribed, and the first author listened to all the interviews multiple times, making necessary corrections to ensure the accuracy of the transcripts. The authors then thoroughly reviewed all the transcribed interviews several times. Following this, we conducted a collective discussion session to collaboratively share our initial interpretations and insights from the data, during which certain patterns were identified.
Coding	The authors re-examined the transcripts and simultaneously color-coded sections representing different codes within the text. Initially, six distinct color codes were applied, but after re-reading and group discussions, some categories were merged, resulting in four remaining color codes.
Generating initial themes	Following the coding process, the authors collaborated to merge the color-coded sections and identify overarching themes for each category. This led to the emergence of four themes.
Reviewing themes	After generating the initial themes, the research group engaged in a discussion to further assess their alignment with TAM2 framework, which is summarized at the end of each theme. The data specifically captured perceptions and beliefs about the current drawbacks and benefits, as well as potential future drawbacks and benefits (Table 2). These findings were then discussed within the group, focusing on the themes in the context of comparing the manual system with the proposed ML system.
Defining and naming themes	The authors engaged in discussions to ensure that the labeling of the themes accurately captured the essence of the data. The names of the themes were revised multiple times until unanimous consensus was reached.
Finalizing the work	Selected data extracts were carefully chosen to illustrate the identified themes. We then compared our findings with existing literature to further explore their implications, which ultimately contributed to the completion of the manuscript.

**Table 2:** Themes and comparison of benefits and drawbacks of manual planning versus the proposed ML system.

Themes	Manual planning		ML system	
	Drawbacks	Benefits	Potential drawbacks	Possible benefits
Controlling PACU throughput	Poor visibility Vulnerability	Better than no planning at all	Possible system miscalculation Not being in control	Enhanced trustworthiness and safety Robust planning Accessibility Lucidity
Prioritizing the patient	Risk of bed overcrowding		Patient confidentiality breach	Patient-focused care Guidance on patients' care duration
Communication strategies	Time consuming	Encourages interpersonal communication	Digital overload	Effective digital information
Adapting new technology			Others have problems with new technology, Technological malfunctions	Management support Building team spirit, User friendly Previous experience eases the transition Tailored education

## Data Availability

The data that support the findings of this study are available on request from the corresponding author. The data are not publicly available due to privacy or ethical restrictions.

## Supporting Information

Supporting Information Appendix 1 comprises the interview guide used in the study. The guide includes 12 questions addressing organizational issues, attitudes toward technology, and specific questions regarding the proposed technological tool.
