# Supplementary material for: Using AI to Predict Patients' Length of Stay: PACU Staff's Needs and Expectations for Developing and Implementing an AI System
Source: J Nurs Manag. 2024 Nov 14;2024:3189531. doi: 10.1155/jonm/3189531 (PMC11925265; doi:10.1155/jonm/3189531)
Supplement: Supporting Information — Appendix 1 comprises the interview guide used in the study. The guide includes 12 questions addressing organizational issues, attitudes toward technology, and specific questions regarding the proposed technological tool. [file 3189531.f1.docx]

Using AI to Predict Patients' Length of Stay PACU Staff’s Needs and Expectations for developing and implementing an AI system

**Appendix 1.**

Interview-guide

Introduction

1. Tell us about your role in the organization.
2. How would you describe the work of planning the patient flow in the postoperative ward today?
3. What pros and cons do you see with how planning is done today?

Attitude to technology

1. What is the role of technology in your work?
2. How do you usually react to new technology? (examples of actions and behaviors).
3. For you, what influences your attitude towards new technology? (Perceived opportunities and/or threats)
4. What are the important prerequisites for new technology to work well for you? (linked to the technology, to the patients, to the organization including colleagues, to own competence, to the actual implementation process).

A hypothetical scenario is presented where the digital planning tool is included. For example, I will now ask several questions about a digital planning tool that would have the purpose of facilitating the planning of the work/patient flow at the postop department. The tool is under development, but we are interested in your needs and expectations of the tool.

The nature of the tool

1. If you think about how planning is done today, could a digital planning tool improve your planning work? If so, how?
2. If you imagine an optimally functioning digital planning tool, what would its features be?
3. Are there particular features or functions that you *don't* want the tool to have?
4. Do you have other concerns related to the introduction of a digital planning tool in the department?

A hypothetical scenario is presented where the digital planning tool is developed and will be introduced on a larger scale in routine practice.

Introduction of the tool

1. What factors do you think are important for a digital tool to be used in routine practice in your department?
